# Prevalence and type of monoclonal gammopathy of undetermined significance in an apparently healthy Nigerian population: a cross sectional study

**DOI:** 10.1186/1471-2326-12-7

**Published:** 2012-06-28

**Authors:** A Lawretta Onwah, Titilope A Adeyemo, Adewumi Adediran, Sarah O Ajibola, Alani S Akanmu

**Affiliations:** 1 Department of Pathology, Federal Medical Centre, Abeokuta, Nigeria; 2 Department of Haematology & Blood Transfusion, Faculty of Clinical Sciences, College of Medicine, University of Lagos, P.M.B. 12003, Marina, Lagos, Nigeria; 3 Department of Haematology & Blood Transfusion, Lagos University Teaching Hospital, Idi-araba, Lagos, Nigeria

**Keywords:** Prevalence, Type, Monoclonal, Gammopathy, Lagos

## Abstract

**Background:**

The prevalence of monoclonal gammopathy of undetermined significance (MGUS), a premalignant plasma-cell disorder has not been determined in our geographic area Nigeria.

**Methods:**

A cross sectional survey was carried on apparently healthy Nigerians selected by multistage sampling technique from the cosmopolitan city of Lagos, Nigeria. Subjects enrolled into the study had 2-step screening for the presence, type and concentration of monoclonal band. Agarose-gel electrophoresis was performed on all serum samples, and any serum sample with a discrete band of monoclonal protein or thought to have a localized band was subjected to Immunofixation. Subjects were also evaluated for Bence jones proteinuria, haematological and biochemical parameters.

**Results:**

Four hundred and ten subjects with a mean age of 45.68 ± 10.3 years, a median of 45.00 years and a range of 20 to 80 years were enrolled into the study. MGUS was identified in only one (0.24 percent) of the 410 study subject. This subject was demonstrated to have a double monoclonal gammopathy; IgGλ at 16.9 g/L and IgAκ at 8.5 g/L. None of them including the sole subject with MGUS had a monoclonal urinary light chain.

**Conclusion:**

Among residents of Lagos, Nigeria, MGUS was found in only 0.24% percent of apparently normal persons with a median age of 45 years. This suggests that MGUS which represents the earliest stage of monoclonal plasma/lymphoid cell proliferation is not a common finding in the relatively young population of Nigeria. Future epidemiologic studies dealing with plasma cell disorders in older people are required to carefully examine the relationship between environmental factors and prevalence of MGUS and its ultimate progression to MM.

## Background

Monoclonal Gammopathy of Undetermined Significance (MGUS) is the most common of a spectrum of diseases called plasma cell dyscrasias [1,2], which are a heterogeneous group of diseases characterized by the expansion of a clone of bone marrow plasma cells that produce monoclonal immunoglobulin (Ig).

The term Monoclonal gammopathy of undetermined significance (MGUS) denotes the presence of a monoclonal protein (M-Protein) in plasma or urine without evidence of multiple myeloma (MM), Waldenström’s macroglobulinaemia (WM), amyloidosis, or B cell related disorders [3]. It has two important characteristics; the first is a plasma immunoglobulin or serum M protein concentration of less than 3 g/dl, only small or no urinary immunoglobulin light chain (Bence jones proteinuria) that has molecular features of the product of a single clone of B lymphocytes or plasma cells and the second is the absence of evidence of an overt neoplastic disorder of B lymphocyte or plasma cells such as lymphoma or multiple myeloma i.e. less than 10% of plasma cells in bone marrow, no anemia, no osteolytic lesions, no hypercalcemia, and no renal dysfunction and most importantly, stability of the M-protein [4].

This condition is also referred to as Essential Monoclonal Gammopathy and Benign Monoclonal Gammopathy but the term Monoclonal Gammopathy of Undetermined Significance (MGUS) remains the preferred designation because of the recognition that this disorder can evolve into a Malignant Monoclonal Gammopathy. While the M- protein may remain stable and benign, it can however progresses to symptomatic MM, Waldenström macroglobulinaemia, light-chain (AL) amyloidosis or a B-cell lymphoma over years of observation in about one-third of patients [5]. Longitudinal follow-up studies have shown that most person with MGUS do not require specific treatment and have a long life span, the subset that evolves to the B cell lymphoproliferative disease will require follow up monitoring and treatment. Because the benign nature of an MGUS is sometimes difficult to ascertain, repeated examinations of the person over long periods are required.

The prevalence of MGUS is reported to vary from 1% to 10% in different series, and the frequency is also reported to increase with age [6-12]. It is a relatively common condition among individuals older than 70 years, occurring in 1 percent of the population older than 50 years, 3 percent in those older than 70 years, and 10 percent in those over 80 years of age. While international frequencies are nearly the same; the prevalence of MGUS has rarely been reported from Africa.

Though it seems important to identify early factors that might predict a malignant transformation in people with MGUS, determining the prevalence of MGUS is undoubtedly a starting point and if there are large number of persons with monoclonal antibody of undefined significance in a given population, it might be an indicator of how prevalent associated B-cell malignancy is in the given population. This study therefore aims to determine the prevalence and type of MGUS in an apparently healthy population of Nigerians.

## Methods

This was a cross-sectional study of apparently healthy adult volunteers. Ethical clearance was obtained from the Lagos University Teaching Hospital Health Research and Ethics Committee (HREC) that approved the study protocol and informed consent documents with reference number; REF.NO. ADM/DCST/HREC/VOL.11/221. Four hundred and fourteen apparently healthy Nigerian adult volunteers without a known liver disease, lymphoproliferative disease, HIV/AIDS, on any immunosuppressant, or organ transplantation were enrolled into the study. Subjects were selected using stratified random sampling of household, with strata defined as the constituent counties or other geographic subdivisions of Lagos, a metropolis. Prospective participants were approached face to face by a trained recruitment coordinator and enrolled once they consent and meet the study enrollment criteria. Not more than one participant was enrolled per household.

A minimum sample size of 386 participants was determined by using the statistical formula of Fisher for calculating sample size N = Z^2^pq/d^2^ when the study population is greater than 10,000 [13]. Standard deviation was set at 1.96 which corresponds to the 95% confidence interval and the proportion in the target population (p) estimated to have MGUS was set at a reasonable estimate of 0.5 (50% - maximum proportion to give the largest minimum sample size). This reasonable estimate was chosen based on the fact that the prevalence of MGUS is yet to be determined for the study population [13]. Degree of accuracy was set at 0.05.

A written informed consent was obtained from each subject and blood sample was taken for the evaluation of hemoglobin concentration, white cell, lymphocyte and platelet count, serum albumin, quantification of total serum protein and albumin, serum creatinine, alkaline phosphatase, presence, size and type of a serum Monoclonal Component, and erythrocyte sedimentation rate (ESR). Urine was also collected for evaluation of the presence of urine monoclonal protein.

Hemogram was carried out on EDTA anticoagulated blood using the sysmex KX 21 N Haematology analyzer. Erythrocyte sedimentation rates in mm/hr were determined manually by the Westergren method. Serum chemistry: Total serum proteins, Serum albumin, serum globulin and serum creatinine were determined by automation. Serum protein electrophoresis (SPEP) was carried out on the subjects using a commercial kit: the Hydragel Protein (E) K20, manufactured by Sebia Inc, Norcross, Ga, USA and designed for seperation of human serum proteins into six major fractions on alkaline buffered (pH 8.5) agarose gel. The M-protein was identified visually as a localized band on the agarose gel electrophoretic strip and as a tall, narrow spike or peak in the β or γ region or rarely, in the α_2_ - globulin area of a densitometer tracing.

Subject identified with a monoclonal band on serum electrophoresis was recalled for evaluation of possible risk factors including family history of MGUS or myeloma, occupational chemical exposure and for screening for urinary Bence -Jones protein by the Bradshaw’s test for Bence -Jones protein^76^ and a bone marrow aspiration to determine the percentage bone marrow plasma cell after obtaining an informed consent. He also had Serum Immunofixation to type and determine the concentration of the monoclonal protein. Serum immunofixation was carried out at Pathcare Laboratories, South Africa by a technique that combines zone electrophoresis with immunoprecipitation.

Data were analyzed using statistical software package: SPSS for windows (version 15; SPSS Inc, Chicago, IL) and Microsoft excel. Results are presented in simple proportions using tables. Comparisons of mean values where applicable, involved the use of student t- test. Discrete variables were compared using chi-square test. The critical level of significance was set at *p* < 0.05.

## Results

A total of 414 participants were enrolled into the study with a mean age of 45.68 ± 10.3 years, a median of 45.00 years and a range of 20 to 84 years. There were 126 (30.4%) females and 288 (69.6%) males with no statistical difference in the mean age of the females (46.33 ± 13 years) with a median age of 47.5 years and that of the males with a mean of 45.39 ± 8.9 years and a median of age of 44 years (*P* = 0.39), (Table 1). While 44.4% of the sample population was in the age range 40- 49 years, only 34% were above 50 years and 1.22% was above 70 years.

**Table 1:** Frequency and mean ages of subjects

**Sex**	**Frequency (%)**	**Mean age (yrs)**	**Median age (yrs)**
Males	288 (69.6)	**45.39 ± 8.93**	44
Females	126 (30.4)	46.33 ± 12.95	47.5
**total**	**414 (100)**	**45.68 ± 10.3**	**45**
**Age (year)**	**Frequency**	**Percent**	
20 -29	24	5.8	
30 – 39	65	15.7	
40 – 49	184	44.4	
50 – 59	104	25.1	
≥ 60	37	8.93	
**Total**	**414**	**100**	

**Figure 1:**
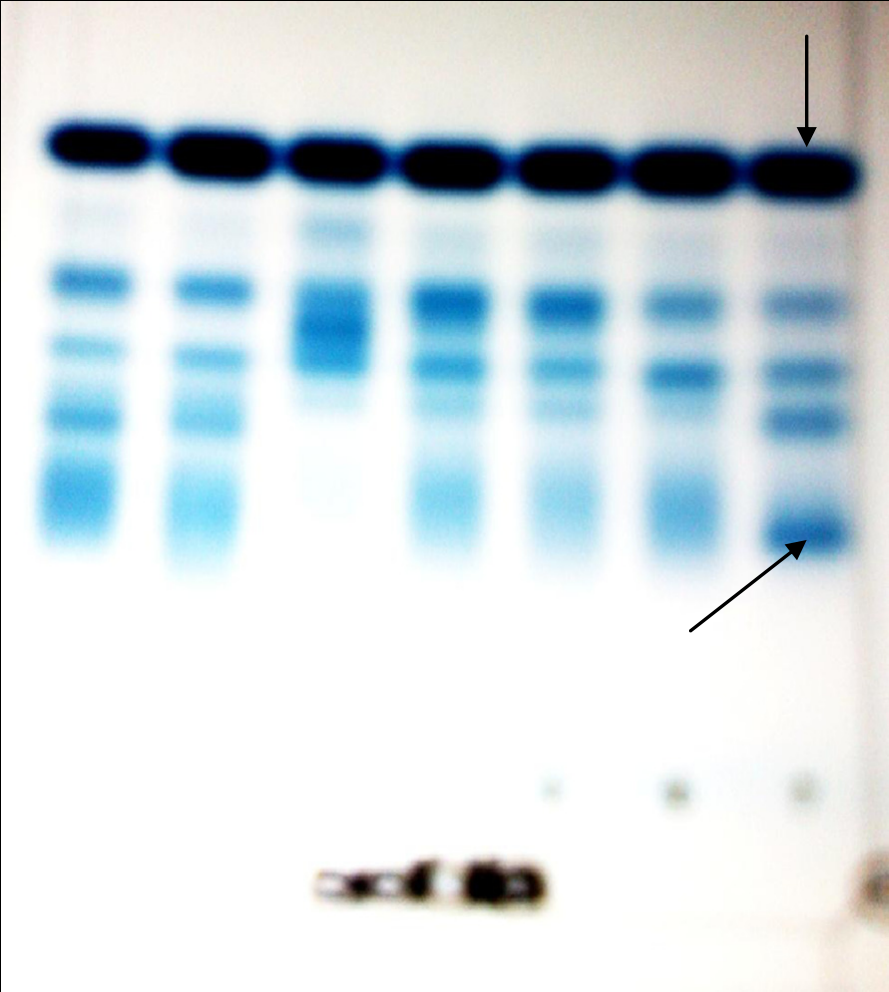
**Serum protein electrophoretic patterns on an Agarose gel showing subject’s β band appearing dense and a discrete band in the γ region.** This is shown with the arrows.

**Figure 2:**
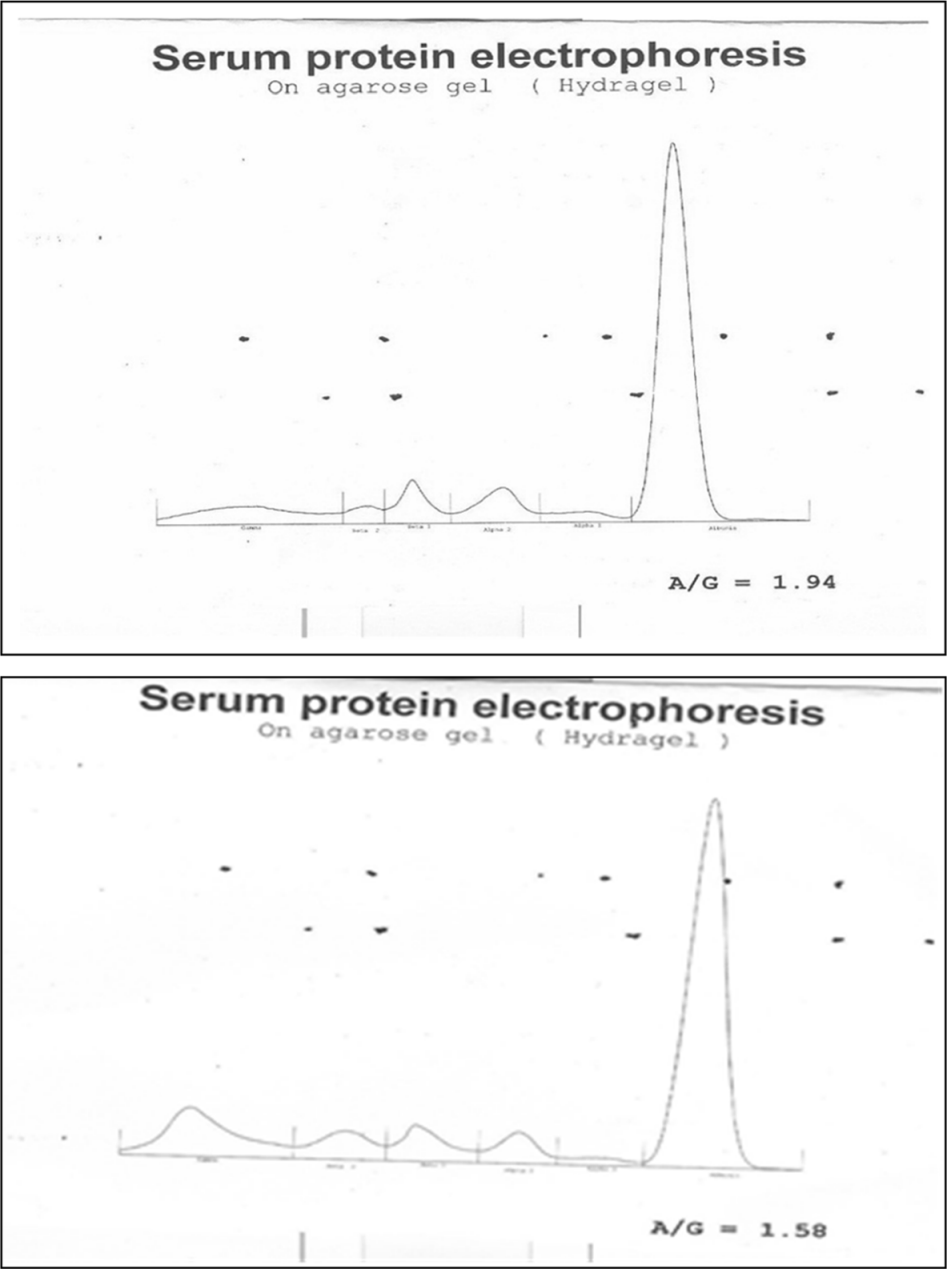
**a: Densitometry tracing of gel electrophoresis of normal serum.b**: Densitometric tracing of gel electrophoresis of subject with monoclonal band.

Four hundred and ten of the 414 subjects enrolled in the study had serum protein electrophoresis (SPEP) done because 4 samples were hemolyzed or insufficient and unsuitable for electrophoretic analysis. Only one of the 410 subjects who had sera subjected to Agarose gel SPEP had the β band appearing dense and a discrete band in the γ region. (Figure 1), Thus only 0.24 percent of the sample population has a monoclonal gammopathy. Figures 2a and 2b shows the densitometry scan of gel of a normal serum and subject with discrete band in the γ region.

### Characteristics of the only study subject with a monoclonal band

**Figure 3:**
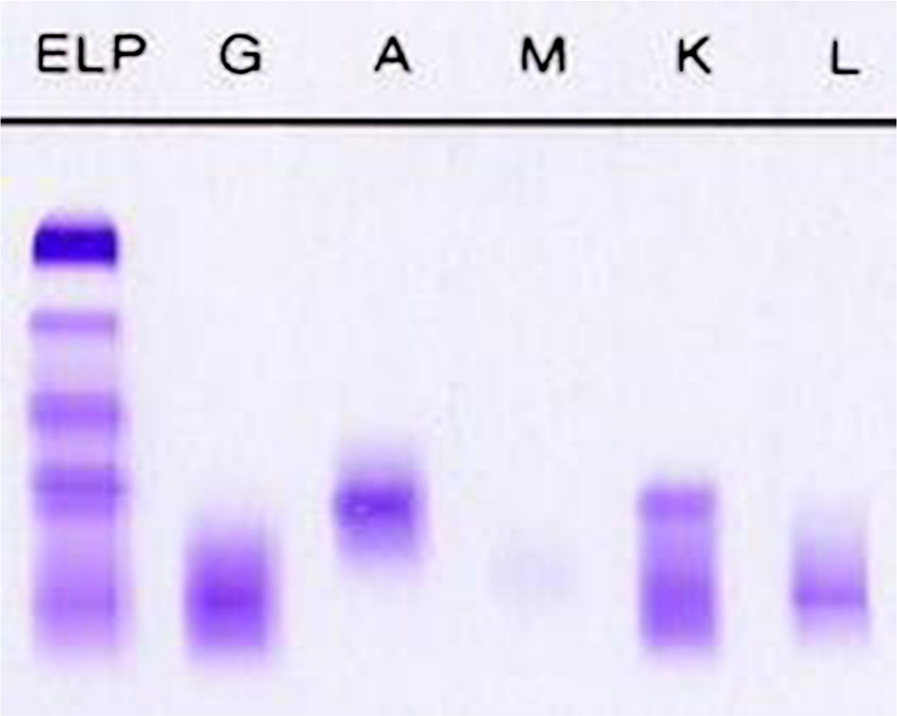
**Serum immunofixation electrophoresis of subject shows two monoclonal bands: IgA kappa (in β2 position) and IgG lambda (in mid-γ region)**.

The sole subject found with a monoclonal band in this study was an apparently healthy 40-year old male, businessman with no family history or occupational risk factor. He had a normal haematological and biochemical profile. ESR was normal at 5 mm/hr. Total serum protein was elevated at 89.27 g/L (range 60-85 g/L) and albumin was normal at 54.67 g/L (range 35-55 g/L) and an A/G ratio of 1.58 (range 1.0-2.1). The beta and gamma fractions were increased. Serum Immunofixation showed two monoclonal peaks IgGλ in mid-γ region and IgA*k* in the β region (Figure 3) with IgG concentration of 16.9 g/l. (Normal range 7.00-16.00 g/L) and IgA of 8.5 g/l. (Normal range 0.70-3.50 g/L). His IgM level was quantified as normal at 0.79 g/l (Normal 0.50-2.50 g/L). Urine was negative for Urinary light chain (Bence jones protein by Bradshaw test.

## Discussion

Monoclonal gammopathy of undetermined significance (MGUS) is one of the most common premalignant disorders in Western countries. Interestingly, prevalence and incidence patterns for MGUS and MM show striking disparity patterns across ethnic/racial groups, most notably the two- to threefold increase in both these disorders in African Americans compared with Caucasians. In contrast, studies on Asian patients show lower prevalence/incidence for MGUS compared with Caucasians.

The prevalence of MGUS is reported to vary from 1% to 10% in different series [6-12]. It occurs in 1 percent of the population older than 50 years and, about 3 percent of the population older than 70 years, and 10 percent in those over 80 years of age. In a population-based study involving 21,463 predominantly white residents of a county in Minnesota, USA, the prevalence of MGUS was 3.2% in persons older than 50 years of age, 5.3% in persons 70 years of age or older, and almost 9% in men older than 85 years of age [6]. In Sweden and France, approximately 3% of persons older than 70 years were found to have an MGUS [14]. The median age at diagnosis of MGUS is generally about 70 years, and less than 2% of people that have been documented to have MGUS are younger than 40 years of age [1]. In the Minnesota study [6], the prevalence of MGUS was found to be higher in men than in women, 4.0% vs. 2.7% among persons that are 50 years or older. This finding has been supported by several other authors [6-12].

The incidence of MGUS has also been reported to be higher in blacks than in Whites [8-10,12]. Cohen et al. [8] reported a prevalence of 8.4% in blacks compared with 3.6% in whites while another retrospective study revealed that the age-adjusted prevalence ratio of MGUS in African American subjects was 3.0 compared with Caucasians [15]. Landgren et al. [10] in their study in Ghanaian men to test for evidence to support an underlying race-related predisposition of a 2-fold higher prevalence of MGUS in African Americans vs. whites found that, compared with white men, the age-adjusted prevalence of MGUS was 1.97 - fold (95% CI, 1.94 - 2.00) higher in Ghanaian men. They concluded that the 2 fold higher prevalence of MGUS in Ghanaian men supports the hypothesis that race-related genetic susceptibility results in the higher rates of MGUS in black populations.

A prevalence of monoclonal gammopathy of 0.24% was found in this study. This contrast the study by Landgren et al. [10] who reported a prevalence of 5.84% of 917 male subjects in the study in Ghanaian men. A possible explanation for this discrepancy is that the subjects in the Ghanaian study were older, with an age range of 50-74 years while in this study, the age ranged 20-84 years with a mean of 45.68 ± 10.3 years. The prevalence of MGUS have been clearly reported to increase with age [6-10]. This figure also contrast the result of Iwanaga et al [11]. from a large study in Nagasaki, Japan who found an overall prevalence of 2.1% in 52,802 survivors of the atomic explosion screened, 1% in participants aged 42-49 years, 1.9% in those 50-59 yrs, 2.6% in those 60-69 yrs, 3% in those 70-79 years and 4.4% in those 80 years and older. Though radiation exposure has been postulated to be a possible predisposing factor for monoclonal gammopathy of undetermined significance (MGUS), the association has remained uncertain.

It is pertinent to note that subject’s age group 40- 49 years constituted the largest age group in our study (44% or 184 of 410 subjects). Only 5 (1.22%) of the subjects were aged 70 years and above and only one of 410 (0.24%) subject had a monoclonal gammopathy and was aged 40 years. The low prevalence rate found in this study when compared with several other studies in black population may also (in addition to relatively younger age of our subjects) be partly due to a smaller sample population size.

The cause of MGUS is not known but certain genetic and environmental exposures are postulated to be risk factors for MGUS. In a report of atomic bomb survivors by Iwanaga et al. [16], those exposed to high levels of radiation at a young age had an increased risk of MGUS. Specific insecticides, pesticides, and fungicides have also been implicated inconclusively in the pathogenesis of MGUS [17]. There is also a genetic element. A study that examined whether monoclonal gammopathy of undetermined significance (MGUS) is increased in first-degree relatives of multiple myeloma (MM) or MGUS patients report that there was an increased risk of MGUS in first-degree relatives (age-adjusted risk ratio [RR], 2.6; 95% CI, 1.9 to 3.4) compared with the reference population [18]. The increased risk seen among relatives of MM (RR, 2.0; 95% CI, 1.4 to 2.8) and MGUS probands (RR, 3.3; 95% CI, 2.1 to 4.8) implies shared environment and/or genetics.

Although MGUS was initially considered a "benign" monoclonal gammopathy, the recognition that this disorder can evolve into a malignant monoclonal gammopathy has led to the use of the more appropriate term "monoclonal gammopathy of undetermined significance. It has recently been demonstrated that virtually all patients with multiple myeloma have a preceding MGUS [19,20]. Landgren et al. [19] showed that in 71 individuals who developed multiple myeloma during a study and in whom serially collected serum samples were obtained from 2 years to 9.8 years prior to the diagnosis of the myeloma. MGUS was present in 100% of patients 2 years prior to the diagnosis of multiple myeloma. At 5 years prior to the diagnosis of multiple myeloma, 95% had MGUS while at 8 or more years prior to the diagnosis of multiple myeloma, 82.4% had a preceding MGUS. The median age of these 71 patients was 70 years and 71.4% were male. The median size of the M protein increased from 0.9 g/dL at 8+ years to 1.6 g/dL at 2 years prior to the diagnosis of multiple myeloma. Approximately one-half of the myeloma patients had a year-by-year increase in M protein until the diagnosis of multiple myeloma. The type of M protein was IgG (68%), IgA (21.5%), IgM (1.5%), or biclonal (3%), and 4.7% had light chain MGUS. Thus, the study established that virtually all patients with multiple myeloma had a preceding MGUS, a finding that was confirmed by another study in which 27 of 30 patients with multiple myeloma had a preceding monoclonal protein [20]. Kristinsson and colleagues [21] found a shorter survival in MGUS patients when compared to the age- and sex-matched normal population. In their report of 241 patients with MGUS, the median survival was 13.7 years, compared to 15.5 years for the USA population.

This study has sought to determine the prevalence rate of MGUS in our population. There is a need for future studies to understand the epidemiology, aetio-biology and identify potential risk factors for MGUS and factors responsible for the progression of MGUS to a serious plasma cell dyscrasia like myeloma in the Nigerian population.

## Conclusion

Among residents of Lagos, Nigeria, MGUS was found in 0.24% percent of apparently normal persons with a median age of 45 years. There is clear evidence from this data that the prevalence of MGUS which represents the earliest stage of monoclonal plasma/lymphoid cell proliferation in Nigerians is low and suggests that MGUS is not a common finding in our relatively young population. Future epidemiologic studies dealing with plasma cell disorders in older people are required to carefully examine the relationship between race, environmental factors and prevalence of MGUS and its ultimate progression to MM. A limitation of this study is that the numbers of participants were relatively small as reflected in the observed prevalence of 0.24% (95% CI, 0.01-1.38). Limited funds did not allow a larger sample size.

## Competing interests

The authors declare that they have no competing interests.

## Authors’ contributions

ALO, TA and ASA were responsible for project design, laboratory work and analysis of results, SOA and AA took part in the laboratory work, ALO, TA, AA and SOA put up criteria in the sorting of patients and contribute to interpretation of results and manuscript write up. ASA was responsible for project supervision. All authors have read and approved the final version of the manuscript.

## Pre-publication history

The pre-publication history for this paper can be accessed here:

http://www.biomedcentral.com/1471-2326/12/7/prepub
